# Translation and Validation of the Malay Revised Second Victim Experience and Support Tool (M-SVEST-R) among Healthcare Workers in Kelantan, Malaysia

**DOI:** 10.3390/ijerph19042045

**Published:** 2022-02-11

**Authors:** Ahmad Zulfahmi Mohd Kamaruzaman, Mohd Ismail Ibrahim, Ariffin Marzuki Mokhtar, Maizun Mohd Zain, Saiful Nazri Satiman, Najib Majdi Yaacob

**Affiliations:** 1 Department of Community Medicine, School of Medical Sciences, Universiti Sains Malaysia, Kubang Kerian, Kota Bharu 16150, Kelantan, Malaysia; drzulfahmi@student.usm.my; 2 Department of Anesthesiology and Intensive Care, School of Medical Sciences, Universiti Sains Malaysia, Kubang Kerian, Kota Bharu 16150, Kelantan, Malaysia; ariffinm@usm.my; 3 Public Health Unit, Hospital Raja Perempuan Zainab II, Kota Bharu 15000, Kelantan, Malaysia; drmaizun@moh.gov.my; 4 Medical Division, Kelantan State Health Department, Kota Bharu 15000, Kelantan, Malaysia; drsaifulnazri@moh.gov.my; 5 Unit of Biostatistics and Research Methodology, School of Medical Sciences, Universiti Sains Malaysia, Kota Bharu 16150, Kelantan, Malaysia; najibmy@usm.my

**Keywords:** second victim experience and support tool, patient safety incidents, reliability, validity, healthcare workers, tertiary care

## Abstract

“Second victims” are defined as healthcare professionals who are traumatized physically, psychologically, or emotionally as a result of encountering any patient safety incidents. The Revised Second Victim Experience and Support Tool (SVEST-R) is a crucial instrument acknowledged worldwide for the assessment of the second victim phenomenon in healthcare facilities. Hence, the aim of this study was to evaluate the psychometric properties of the Malay version of the SVEST-R. This was a cross-sectional study that recruited 350 healthcare professionals from a teaching hospital in Kelantan, Malaysia. After obtaining permission from the original author, the instrument underwent 10 steps of established translation process guidelines. Pretesting of 30 respondents was performed before embarking on the confirmatory factor analysis (CFA) to evaluate internal consistency and construct validity. The analysis was conducted using the R software environment. The final model agreed for 7 factors and 32 items per the CFA’s guidelines for good model fit. The internal consistency was determined using Raykov’s rho and showed good results, ranging from 0.77 to 0.93, with a total rho of 0.83. The M-SVEST-R demonstrated excellent psychometric properties and adequate validity and reliability. This instrument can be used by Malaysian healthcare organizations to assess second victim experiences among healthcare professionals and later accommodate their needs with the desired support programs.

## 1. Introduction

Healthcare professionals often work in high-stakes, tense working environments in which unwelcomed, negative patient outcomes are inevitable. Even with meticulous and highly disciplined work ethics, patient safety incidents (PSIs) can only be minimized and are not completely avoidable. The slightest error or mildest incidence of substandard care can inflict harm on patients. This then provokes a chain reaction for the involved healthcare professionals.

The term “second victim” was initially coined by Albert Wu [1]. The first victims are patients and their families, and the second victims are healthcare providers (HCP) attending to the needs of the first victims. Formally defined, this situation is acknowledged as the second victim syndrome (SVS): HCPs who commit an error and are traumatized by the event can manifest psychological (embarrassment, frustration, guilt, remorse, fear of future errors, anxiety, grief, anger, and depression) [2,3,4,5,6,7,8,9,10,11,12], cognitive (compassion dissatisfaction, burnout, secondary traumatic stress, and troubling memories) [4,13,14,15,16], and/or physical reactions (sleeping difficulties; frequent nausea; increase in blood pressure, heart rate, or respiratory rate) [3,17,18,19]. Recent articles do not limit SVS to result only from medical error; instead, they can result from a PSI, which can be any event or circumstance that could have resulted, or did result, in unnecessary harm to a patient [20]. PSIs can be adverse events, near misses, or any kind of morbidity and mortality [21,22]. 

The prevalence of SVS ranges from 9% to 90% among HCPs [8,23,24,25,26,27,28,29,30,31]. This discrepancy occurs due to the different nature of occupational settings. Higher-risk disciplines, such as surgical-based fields, obstetrics, and gynecology, have higher odds of SVS compared with other medical disciplines. In addition, SVS is synonymous with dysfunctional coping strategies, including practicing defensive medicine [32,33,34], post-traumatic stress disorder [35,36], increased turnover rate and absenteeism [37,38], or worse, committing self-inflicted injury or suicide [39]. Furthermore, the consequences of SVS are not limited to the affected HCPs; however, sequelae can be deleterious, as manifested by future sub-optimal care [40] and repercussive clinical errors [41,42,43]. Furthermore, the domino effect of SVS can be spilled onto third victims, the organization or hospital itself, via reputational, legal, or economic issues [44,45,46]. Thus, SVS has been identified as an important issue in patient safety by experts [47] and political stakeholders [48].

In addition, the root cause of SVS can be attributed to a poor healthcare organizational culture. Healthcare organizational culture, defined as how the people of an organization interact within the system, share their thinking, and behave to improve the quality of care or make it easier to be known as just culture, is not widely appreciated among healthcare organizations [49,50]. The counterstatement of just culture is the stigma of being judged or the punitive culture of healthcare organizations [39,51]. Given Malaysia’s fairly moderate cultivation of just culture, there is still vast room for improvement [52,53]. To embark on a clear situational analysis of SVS and provide solutions, a valid assessment tool is necessary. 

There are a few notable instruments available to assess SVS [17,25,54,55]; however, the most widely recognized is the Second Victim Experience and Support Tool (SVEST), published by Burlison in English [56]. The SVEST has been validated and translated into Korean [57], Iranian [58], Italian [59], Spanish (Argentina) [60], Chinese [61], Danish [62], and Spanish (Spain) [63]. Since 2020, an improvised revision of the SVEST (SVEST-R) has been made available in English [64] and subsequently translated into German [65]. Even though the initial English version focused on pediatric clinical care and the revised version on the neonatal care unit, other translations have broadened this scale’s applicability to various clinical departments, teaching hospitals, and multiple healthcare professions, thus enhancing the generalizability and usability of the questionnaire. Furthermore, using a well-validated tool would then bolster the tool’s future application to second victim-related studies and offer better management strategies for healthcare professionals upon encountering any PSI in the long run.

As regards Malaysia, no publications have studied SVS, and no valid and reliable instrument has been created with the intention of addressing SVS. Therefore, the present study evaluated the psychometric properties of the Malaysian version of the SVEST-R (M-SVEST-R) and focused on determining the levels of SVEST-R competence among HCPs in Malaysia.

## 2. Materials and Methods

### 2.1. Study Design and Participants

A cross-sectional study was conducted between June and August 2021 in a teaching hospital in Kelantan, Malaysia. The study adopted a multiple-step standard approach to translating, culturally adapting, and testing the questionnaires. The respondents were HCPs (doctors, nurses, and assistant medical officers) who were working in clinical care, had previously engaged in any form of PSI within the last 5 years, and self-admittedly did not possess any psychiatric-related illnesses. For the recruitment process, initially, a list of total HCPs in the hospital was gathered with the aid of administrative officials and coresearchers. Next, the respondents were chosen by systematic random sampling from the list. The questionnaires were delivered online and did not require any face-to-face meetings. The respondents were required to determine whether they met the inclusion criteria first and to consent to enrolment. Then, the respondents were permitted to fill out the rest of the questionnaires.

### 2.2. The SVEST-R Instrument

The SVEST scale, made of a self-reported questionnaire, was originally developed after an extensive literature review, group discussions among experts in patient safety, and the second victim concept [56]. Since its conception in 2017, the SVEST questionnaire has been translated from the original English version into other languages. The original SVEST questionnaire has seven dimensions with two negative outcome variables. The seven dimensions include psychological distress (4 items), physical distress (4 items), colleague support (4 items), supervisor support (4 items), institutional support (3 items), non-work-related support (2 items), and professional self-efficacy (4 items). The two negative outcome variables are turnover intentions (2 items) and absenteeism (2 items). All items are close-ended questions ranked based on a five-point Likert scale ranging from 1 (strongly disagree) to 5 (strongly agree). High SVEST scores indicate a high prevalence of second victim responses, a perception of insufficient support resources, and a high magnitude of negative work outcomes. Later, it was revised into a 35-item SVEST-R questionnaire, in which the work-related support dimension was omitted, and a positive outcome dimension, namely, resilience, was introduced [64]. 

Good construct validity was reported for the SVEST-R (chi-square test x^2^ = 1555, degree of freedom (DOF) = 524, root mean square error of approximation (RMSEA) = 0.079, comparative fit index (CFI) = 0.821, and standardized root mean squared residual (SRMR) = 0.091). Factor loadings of all items ranged from 0.42 to 0.92, while Cronbach’s alpha ranged from 0.66 for colleague support to 0.86 for physical distress. 

### 2.3. Translation and Cultural Adaptation

To begin, the corresponding author contacted the original author of the SVEST-R via e-mail. Cordially, the original author gave permission and authorization for the translation of the SVEST-R into the Malay language.

The standard process of translation and cultural adaptation followed the guidelines set forth by Wild [66]. This step-by-step guideline was made up of 10 stages:

#### 2.3.1. Preparation

Before embarking on the translation, the preparation stage included finding the translators, briefing them regarding the research, and setting deadlines for each stage.

#### 2.3.2. Forward Translation

The forward translation stage involved hiring two independent translators: a medical doctor with a public health background (translator 1) and a teacher from the Linguistics Department of Universiti Sains Malaysia (USM; translator 2). Translator 1 was a healthcare worker and had good knowledge regarding patient safety issues, while translator 2 was a native Malay speaker and concurrently a professional translator with vast experience in translating medical background instruments. They conversed well and were fluent in both English and Malay.

Both translators conducted the translation from English to Malay independently, and any difficult or confusing words, items, or terms were highlighted.

#### 2.3.3. Reconciliation

The results of both independent forward translations were compared and then merged into a single translation. Experts working in patient safety, hospital quality and management, teaching, clinical care, and counseling (Table 1) were recruited to review the single translation. Words, phrases, or terms that were confusing or irrelevant with local context were substituted and adapted with more suitable words or phrases. In addition, the content and face validity processes were also run during this stage to further check the concept and item adequacy.

#### 2.3.4. Back Translation

After producing the reconciled single Malay translation, it was translated back to English to compare it with the original English version. Two translators were hired, and both translators shared similar criteria as translators 1 and 2 in the forward translation stage. A fortnight was allocated to producing the back translation.

#### 2.3.5. Back Translation Review

Once the back translation was ready, it was scrutinized by the same review committee for any discrepancies with the original English version. The committee assessed the equivalence, either conceptual, by item, or semantic, of both translated versions. Overall, the review committee mutually consented to some minor adjustments to the back translation.

#### 2.3.6. Harmonization

The harmonization stage sought further agreement between the reconciled Malay translated version and both the back-translated and original versions. This stage ran a quality control check to improve any undue discrepancies. The review committee concluded that the reconciled Malay translated version was apt to be the prefinal version of the M-SVEST-R.

#### 2.3.7. Cognitive Debriefing

Cognitive debriefing functioned as a test run for the prefinal version using a conveniently selected small group of respondents (five medical officers and five nurses). They shared similar inclusion criteria as the main respondents: they worked in clinical services, did not possess any psychiatric-related illnesses, and had not engaged in any kind of PSI within the last 5 years. Cautiously briefed beforehand, they were given the prefinal version of the M-SVEST-R to complete. From this session, a few difficult words were found to be difficult to comprehend and confusing. The respondents were asked for their opinions and recommendations for further improvements to the translation.

#### 2.3.8. Review of Cognitive Debriefing Results and Finalization

The findings of the cognitive debriefing stage were reviewed. The findings and recommendations were analyzed, and amendments were made accordingly by the review committee.

#### 2.3.9. Proofreading

The proofreading stage aimed to polish and consolidate the final version of the M-SVEST-R. Any grammatical errors or typographical errors were recognized. Lastly, the review committee conducted a final check to see whether any further adjustments or corrections were needed.

#### 2.3.10. Final Report

A final report on the entire process was properly created. Each correction and all changes applied were explained in words, as this is essential as a future reference.

### 2.4. Pretesting

Pretesting was performed at the teaching hospital. Using a convenient selection method and similar inclusion criteria, 30 HCPs (medical officers, nurses, and assistant medical officers) were recruited to participate. This was a further test-run process to check for any possible shortfalls in the translated questionnaire. Because this study was conducted during the COVID-19 pandemic, pretesting was also conducted online. An online form coupled with an introductory video was created to brief the respondents about the details of the study.

### 2.5. Data Analysis

Data analysis was performed using R software [67]. Confirmatory factor analysis (CFA) was conducted using lavaan and semTools of R packages [68,69]. 

A robust maximum likelihood was preferred [70]. Descriptive statistics for the M-SVEST-R were computed, as explained by Burlison [56]. Mean scores were measured for every item, dimension, and outcome. In addition, the percentage of agreement was introduced according to the number of participants who achieved a mean score ≥ 4.0 (a proxy that shows that a negative outcome for each dimension had occurred due to a second victim experience).

Construct validity was assessed using CFA. Standardized factor loadings were calculated to determine the dimensionality of the scale, and a factor loading > 0.40 was accepted. The goodness of fit of the model was assessed using SRMR, RMSEA, CFI, and chi-squared test. The model was considered “relatively good” if the following criteria were met: a cut-off value for CFI > 0.90, a cut-off value for the chi-square statistic divided by the DOF < 3, and cut-off values for the SRMR and RMSEA < 0.08 (adequate fit) [71].

The model revision was considered based on factor loadings, standardized residuals (SRs), modification indices (MIs), and the theoretical background. Parameters with an SR ≥ 2.58 or an MI ≥ 3.84 were considered for possible changes in the model’s specifications. If there was a correlation r ≥ 0.85, multicollinearity was expected. To compare the models, the Akaike information criterion (AIC) and the Bayesian information criterion (BIC) were applied. Models with lower AIC and BIC values were selected as the best-fitting mode of CFA [70]. 

As regards the reliability assessments, internal reliability consistency was determined by Raykov’s rho coefficient, whereby a threshold of ≥0.7 was considered adequate [71]. Regarding the CFA, the recommendation was to acquire a ratio of 1:10, meaning an item per 10 respondents. Therefore, because the M-SVEST-R acquires 35 items, a total of 385 respondents were needed with the inclusive addition of a 10% nonresponse rate [72].

### 2.6. Ethical Considerations

The study was approved by the Medical Research and Ethics Committee (MREC) of the Ministry of Health (NMRR-21-171-58022) and the Human Research Ethics Committee of Universiti Sains Malaysia (USM/JEPeM/21020161). Data confidentiality was sternly preserved. Data were only restricted to the authors and supervisors. Then, reporting and publication were performed anonymously without requiring any personal identification.

## 3. Results

### 3.1. Demographic Characteristics of Respondents

At the beginning, 403 HCPs were registered, and 370 of them confessed that they had experienced at least one PSI episode within the last 5 years. Out of these eligible participants, 20 HCPs were unable to complete all the questionnaires and had some items missing from the data. Finally, 350 participants were considered by this research, which yielded a 94.5% response rate.

The participants comprised 324 nurses (93%), 24 medical doctors (6.3%), and 2 assistant medical officers (0.7%), of whom 302 were women (86.3%) and 304 (87%) were married. The mean age of the participants was 35.2 years, with 12 years of working experience.

According to the respective departments, 88 participants (25.1%) were from anesthesiology and intensive care, 84 participants (24%) were from pediatrics, 49 participants (14%) were from surgery, 46 participants (13.1%) were from internal medicine, 22 participants (6.3%) were from obstetrics and gynecology, 21 participants (6%) were from orthopedics, and 40 participants (11.7%) were from others (Table 2).

### 3.2. Domain Descriptive Findings

The mean scores ranged from 2.06 (standard deviation (SD): 0.75) for colleague support to 2.84 (SD: 0.76) for institutional support. As regards the percentage of agreement (SVEST scores), the values ranged from 1.4% (colleague support) to 11.4% (psychological distress). These percentages reflected that 1.4% of the respondents felt that they had weak support from colleagues, while 11.4% suffered from psychological distress after experiencing PSIs. Despite good colleague support, there was a relatively marked prevalence of a lack of supervisor support (11.1%), and a total of 14.2% of the respondents disclosed negative outcomes (turnover intention and absenteeism). Otherwise, 8.9% of the respondents did increase their resilience after being involved in any PSIs. A summary of the findings is illustrated in Table 3. 

### 3.3. Psychometric Validation of the M-SVEST-R

During the harmonization part of the translation process, the appointed expert panel reviewed all the items and proved them to be relevant and adequately comprehensive. The item level of CVI (I-CVI) and the scale level of CVI (S-CVI) both scored >0.8, which were considered good [73].

To select the model with the best fit, four models were discussed throughout the process. The initial model, model 1, started with 9 factors with 35 items, the same model as prescribed by the original SVEST-R. The majority of the items scored >0.60, except for 3 items that had inadequate standard factor loadings (item factor loading <0.41 or >1) [72]. Specifically, items 11 (“My colleagues help me feel that I am still a good healthcare provider despite any mistakes I have made*”) of the colleague support factor, 16 (“My supervisor blames individuals”) of the supervisor support factor, and 20 (“Concern for the well-being of those involved in these situations is not strong at my organization”) of the institutional support factor revealed poor factor loadings of less than 0.41. 

Model 2 shared similar factors and items with model 1 but applied modified indices. However, the factor loadings did not differ significantly. As regards model 3, this model removed 3 underperforming items (11, 16, and 20) and satisfied 32 items with the existing 9 factors. The result was remarkable, as all the factor loadings were >0.60. Although an acceptable threshold of factor loadings was achieved, the analysis found a mis-specification error. The mis-specification error rose due to high multicollinearity (>0.9) between the factors of psychological and physical distress, turnover intention, and absenteeism [74].

To solve the above issues, model 4 was created by introducing 2 new combined factors: distress (combination of psychological and physical distress) and negative outcomes (combination of turnover intention and absenteeism). Eventually, model 4 was composed of 7 factors and 32 items (deleting items 11, 16, and 20) with satisfying factor loadings (>0.6). Table 4 explains the evolving factor loadings for each model.

Table 5 shows a summary of the fit indices for the suggested models. As regards the fit indices, models 1 and 2 clearly did not achieve the standard values, with poor factor loadings for the 3 aforementioned items. Despite attaining the targeted fit indices, model 3 sustained a mis-specification error, as described above. Model 4 successfully achieved a reasonable fit for the M-SVEST-R indicated by the CFA (x^2^ = 797, DOF = 418, *p*-value < 0.0001, RMSEA = 0.051, CFI = 0.946, TLI = 0.935, and SRMR = 0.055).

Table 6 explains the evolving Raykov’s rho cross-cutting of the four studied models. For model 4, Raykov’s rho coefficients were overall good, as all estimates exceeded >0.70 except for the institutional support factor (0.68). Overall, the total rho scale was reasonably acceptable at 0.83.

## 4. Discussion

Quantitative measurements were used to determine the occurrence of second victim experiences in Malaysian healthcare environments, the degree of support offered, and the manifested work-related outcomes deemed to be detrimental issues [28,75]. This study aimed to examine the reliability and validity of the M-SVEST-R in accordance with validated translation and psychometric methods used by the original authors [56,64]. The standard methods were assessed for content validity, internal consistency, and construct validity using CFA [13,75]. In view of the different cultures, healthcare delivery systems, and countries, the extent of the second victim phenomenon should be observed on a case-by-case basis due to the unique presentation of each setting. Therefore, this study offers important findings regarding the psychometric properties of the M-SVEST-R for future use in the Malaysian healthcare setting.

During the translation process, the experts were in agreement regarding the content and the number of domains and items of the M-SVEST-R. The contents were apt and technically feasible for use as questions, as proven by the CVI score. None of the experts objected to the number of domains and items. However, the reconciliation period revealed a few minor adjustments to wording preference and technical arrangements for the online form. Otherwise, the instructions, with the addition of a 2-minute introductory video, were simple and easily understood. In addition, a five-point ordinal Likert scale was considered appropriate and sufficient for capturing the intended responses.

In addition, pretesting drew a few pieces of feedback regarding the form’s arrangements and the introductory video. For example, the item arrangement was more user-friendly when using tick symbol options. Furthermore, the time lapses in the introductory video were quite fast, and some respondents were unable to comprehend the whole idea of the study and needed a few replays to catch the information. Hence, adjustments were applied accordingly.

In view of the agreement percentage, there was no factor that exceeded a mean of 3, with the highest being institutional support at 2.84. In view of this agreement, the highest was 11.4% for psychological distress, and the lowest was 3.4% for colleague support. The findings for psychological distress were in accordance with similar studies in Denmark, Argentina, and Iran. In view of the type of support, colleague support proved to be the most preferred type of support, as formerly produced in the original SVEST, original SVEST-R, Iranian version P-SVEST, and Danish version D-SVEST [56,58,60,62,64].

The analysis initially considered four models, from models 1 to 4. Models 1 and 2 (with modification indices), which retained the original 35 items and 9 factors, did not achieve the intended fit indices and good factor loadings, as revealed in Table 4 and Table 5. Poor factor loadings recorded were from 3 items (11, 16, and 20).

In support of this, the Argentinean version of the SVEST had similarly deleted item 16, “My supervisor blames individuals.” On a similar note of encountering faulty translation, the Chinese version C-SVEST and Danish version D-SVEST retained this item but with rephrasing adjustment [57,60,61,62]. Meanwhile, concerning item 11, “My colleagues help me feel that I am still a good healthcare provider despite any mistakes I have made, *” it was formulated as a negative statement item, rendering difficulties in comprehension and giving responses. Similar difficulties were also portrayed in the translation of the Danish SVEST [62]. As regards item 20 (“Concern for the well-being of those involved in these situations is not strong at my organization”), other translations did not encounter any issues and retained the item. Aside from item 20, 2 other items in the institutional support domain (18 and 19) used positive connotations in addressing institutional support. The reason was perhaps item 20 used negative connotations, and consequently, the idea was not appreciated and caused insecurity in responses, as respondents would have to testify against their own institution [76]. Omitting these 3 poorly performing items might have led to a deficiency in the findings, especially as the instrument was tested for validation only at a single institution. However, thankfully, there were other positive statement items in the same domain that shared the same meaning as these deleted items, possibly retaining the overall objectives of the domain. Furthermore, if the items were not deleted, the fit indices would have remained poor and would have been unable to achieve the intended target.

Model 3 (deleting the 3 items) revealed good factor loadings and excellent fit indices, but surprisingly came across an error specifically regarded as a mis-specification error. Mis-specification errors occur when there is high multicollinearity (correlation of more than 0.85) among the factors [71,74]. High multicollinearity was recorded between psychological distress and physical distress, as well as turnover intention and absenteeism. 

To curb the situation, model 4 was created by transforming psychological distress and physical distress into a single factor of distress, and turnover intention and absenteeism were turned into the negative outcome factor. Merging psychological and physical distress was emphasized by the Chinese version C-SVEST, as nurses tend to mask their physical distress with emotional or psychological symptoms. In other words, they psychosomatize their mental stress, and consequently, there is no clear distinction between these two types of distress [77]. A similar situation was previously replicated in the K-SVEST, whereby they combined turnover intention and absenteeism into a factor: negative work-related outcome [57]. Understandably, the Chinese and Korean versions of the SVEST provided insight into the cultural differences between the Eastern and Western versions of the SVEST. Model 4 also abandoned the three poor-performing items and eventually produced a model with 32 items and 7 factors. Model 4 successfully achieved good factor loadings for each item (>0.65) and acceptable fit indices (chi square = 797, DOF = 418, *p*-value < 0.0001, RMSEA = 0.051, CFI = 0.946, TLI = 0.935, and SRMR = 0.055). 

As regards Raykov’s rho, the total scale rho was considered good at 0.83 (ranging from 0.68 to 0.93). Other SVEST versions utilized internal consistency based on Cronbach’s alpha instead of the composite reliability of Raykov’s rho. 

Cronbach’s alpha is widely popular for reliability studies among researchers. Despite its popularity, Cronbach’s alpha has been criticized, as it assumes unidimensionality and that all items are equally constructed with the factors. In contrast, Raykov’s rho considers multiple factors and the difference in factor loadings among items [78,79,80]. Cronbach’s alpha for the original SVEST was 0.79, the Korean version was 0.71, the Argentinian version was 0.805, the Iranian version was 0.76, the Danish version was 0.91, the Italian version was 0.88, the revised SVEST-R was 0.86, the Chinese version was 0.866, and the German version of the SVEST-R was 0.884 [56,57,58,59,60,61,62,64,65]. Even though institutional support was the sole factor contributing to Raykov’s rho of less than 0.70, the score was considered adequate when compared with Cronbach’s alpha of the original SVEST (0.64), the K-SVEST (0.59), and the Danish version of the SVEST (0.68) [56,57,62]. 

Removing the 3 poor-performing items for factor loadings increased the total rho scale, as described in Table 4. As mentioned, due to the mis-specification error and solution to combine factors, model 4 fulfilled consistent good factor loadings, fit indices, and Raykov’s rho.

Compared with earlier studies that involved only nurses as the respondents, this study, despite involving nurses, also involved doctors and assistant medical officers in the sampling population. However, nurses still made up the majority of the respondents. The response rate among doctors was low, possibly due to the time constraints caused by having to manage COVID-19. In addition, the respondents belonged to various departments and were not confined to certain clinical departments, such as pediatrics or the intensive care unit, as in previous studies. Looking into heterologous departments, perhaps the generalizability and representativeness of the data are the additional value offered by this study.

In addition to these strengths, limitations were also addressed. The majority of the Malaysian population can converse well in the Malay language, as it is Malaysia’s national language. Notwithstanding this importance, during the process of translation from English to Malay, linguistic and administrative issues were encountered [81]. For instance, there were a few medical terms or concepts that were rather complicated to comprehend for non-medical-background translators. In addition, few linguistically trained medical personnel are engaged in the translation process.

Next, as mentioned, this study deployed online-based questionnaires distributed via e-mail and an online messaging platform. Undeniably, online questionnaires are flexible, cost-effective, easy to set and distribute to respondents, robust in terms of transfer errors, and most importantly, effective at mitigating safety concerns and ensuring less direct contact, especially given the COVID-19 pandemic [82]. However, online questionnaires come with a limitation. Due to a lack of verbal and direct one-way communication, the respondents could have been motivation-deprived or found it difficult to comprehend the intention of this study, even though the authors had offered a 2-minute introductory video regarding the study.

Furthermore, the stigma of being blamed and the punitive culture of the Malaysian healthcare environment are confounding factors in this study. Certain questions were possibly not appreciated by some respondents, as they could have felt fearful of negative responses, even though the survey promised confidentiality and anonymity [76,83].

Lastly, this study did not aim to provide an overall assessment of SVS in Malaysia. Future studies should be conducted with more diverse respondents and clinical settings. However, the descriptive findings of the study can still be utilized as the foundation for later studies.

## 5. Conclusions

In conclusion, the M-SVEST-R questionnaire is a valid and reliable instrument for assessing SVS and its effects on the healthcare setting of Malaysia. Future second victim-related studies are encouraged to utilize this instrument. The instrument can provide insight for a better support system and ways to provide the best care, not only for second victims, but also for the overall chain of healthcare services, including patients and organizations themselves.

## Figures and Tables

**Table 1:** Occupation, years of experience, area, and academic level of the experts.

	Position	Years of Professional Experience	Area	Academic Level
Expert 1	Hospital quality officer	20 years	Patient safety, hospital quality, and management	Medical graduate Postgraduate in cardiac anesthesiology Certified for hospital quality management and business architecture
Expert 2	Medical director	20 years	Patient safety, hospital quality and management	Medical graduate Postgraduate in public health
Expert 3	Anesthesiologist	10 years	Teaching and clinical care	Medical graduate Postgraduate in anesthesiology
Expert 4	Psychiatrist	10 years	Teaching and clinical care	Medical graduatePostgraduate in psychiatry
Expert 5	Counsellor	10 years	Teaching and clinical care	Medical graduate Postgraduate in psychology
Expert 6	Counsellor	10 years	Counselling and therapy	Psychology graduate
Expert 7	Occupational health doctor	5 years	Clinical care	Medical graduate Certified for occupational health
Expert 8	Medical lecturer	10 years	Teaching	Medical graduate Postgraduate in medical education
Expert 9	Head of nurse	20 years	Clinical care	Nursing diploma

**Table 2:** Characteristics of respondents (n = 350).

Characteristics (n = 350)	N (%)	Mean (SD)
Gender		
Male	48 (14%)	
Female	302 (86%)	
Age (years)		35.2 (7.35)
Race		
Malay	338 (97%)	
Non-Malay	12 (3%)	
Marital status		
Married	304 (87%)	
Single	46 (13.1%)	
Current department		
Anesthesiology and critical care	88 (25%)	
Pediatrics	84 (24%)	
Surgery	49 (14%)	
Internal medicine	46 (13%)	
Obstetrics and gynecology	22 (6.3%)	
Orthopedics	21 (6%)	
Others	40 (11.7%)	
Working experience (years)	12 (7.4)	
Position		
Nurses	326 (93%)	
Medical officers	17 (4.9%)	
House officers	5 (1.4%)	
Assistant medical officers	2 (0.6%)	

**Table 3:** Domain descriptive findings of the M-SVEST-R (n = 350).

Domain	Agreement (%)	Mean	SD
Psychological distress	11.4	2.48	1.10
Physical distress	3.7	2.22	0.85
Colleague support	1.4	2.06	0.75
Supervisor support	11.1	2.75	0.87
Institutional support	6.6	2.84	0.76
Professional efficacy	6.0	2.23	0.97
Turnover intention	7.1	2.06	1.04
Absenteeism	7.1	2.20	1.03
Resilience	8.9	2.22	1.02

SD: standard deviation.

**Table 4:** Confirmatory factor analysis: standardized factor loadings for the M-SVEST-R (n = 350).

	Factor Loadings			
Domains and Items	Model 1	Model 2	Model 3	Model 4
Distress				
(a) Psychological Distress				
1. I have experienced embarrassment from these instances.	0.81	0.744	0.809	0.742
2. My involvement in these types of instances has made me fearful of future occurrences.	0.729	0.773	0.729	0.727
3. My experiences have made me feel miserable.	0.89	0.835	0.891	0.83
4. I feel deep remorse/guilt for my past involvement in these types of events.	0.827	0.821	0.827	0.8
(b) Physical Distress				
5. The mental weight of my experience is exhausting.	0.835	0.829	0.835	0.838
6. My experience with these occurrences can make it difficult to sleep regularly.	0.793	0.793	0.793	0.792
7. The stress from these situations has made me feel queasy or nauseous.	0.829	0.793	0.829	0.8
8. Thinking about these situations can make it difficult to have an appetite.	0.848	0.826	0.848	0.827
9. I have had bad dreams as a result of these situations.	0.796	0.792	0.796	0.787
Colleague Support				
10. My colleagues can be indifferent to the impact these situations have had on me.	0.704	0.712	0.704	0.705
11. My colleagues help me feel that I am still a good healthcare provider despite any mistakes I have made *	−0.017	−0.021	Deleted	Deleted
12. My colleagues no longer trust me.	0.78	0.766	0.78	0.776
13. My professional reputation has been damaged because of these situations.	0.848	0.87	0.848	0.85
Supervisor Support				
14. I feel that my supervisor treats me appropriately after these occasions *	0.714	0.655	0.714	0.656
15. My supervisor’s responses are fair *	0.811	0.803	0.804	0.794
16. My supervisor blames individuals.	0.088	0.042	Deleted	Deleted
17. I feel that my supervisor evaluates these situations in a manner that considers the complexity of patient care practices *	0.731	0.784	0.737	0.779
Institutional Support				
18. My organization understands that those involved may need help to process and resolve any effects they may have on care providers *	0.793	0.823	0.801	0.832
19. My organization offers a variety of resources to help get me over the effects of involvement with these instances *	0.617	0.592	0.617	0.6
20. Concern for the well-being of those involved in these situations is not strong in my organization.	−0.19	−0.19	Deleted	Deleted
Professional Self-Efficacy				
21. Following my involvement, I experienced feelings of inadequacy regarding my patient care abilities.	0.688	0.68	0.688	0.687
22. My experience makes me wonder if I am not really a good healthcare provider.	0.785	0.818	0.786	0.818
23. After my experience, I became afraid to attempt difficult or high-risk procedures.	0.764	0.748	0.764	0.763
24. These situations have negatively affected my performance at work.	0.809	0.809	0.808	0.82
Negative Outcomes				
(a) Turnover Intention				
25. My experience with these events has led to my desire to take a position outside of patient care.	0.829	0.765	0.829	0.779
26. Sometimes, the stress from being involved with these situations makes me want to quit my job.	0.824	0.817	0.824	0.841
27. I have started to ask around about other job opportunities.	0.839	0.773	0.839	0.765
28. I plan to leave my job in the next 6 months because of my experience with these events.	0.763	0.716	0.763	0.722
(b) Absenteeism				
29. My experience with an adverse patient event or error has resulted in me taking a mental health day.	0.705	0.632	0.705	0.683
30. I have taken time off after one of these instances occurs.	0.78	0.693	0.78	0.738
31. When I am at work, I am distracted and not 100% present because of my involvement in these situations.	0.849	0.803	0.849	0.867
(c) Resilience				
32. Because of these situations, I have become more attentive to my work *	0.847	0.624	0.847	0.68
33. These situations have caused me to improve the quality of my care *	0.734	0.757	0.734	0.78
34. My experience with an adverse patient event or error has resulted in positive changes in procedures or care on our unit *	0.716	0.881	0.716	0.861
35. I have grown as a professional as a result of an adverse patient event or error *	0.845	0.771	0.845	0.697

For model 4, the distress factor combined psychological and physical distress, and the negative outcomes factor combined turnover intention and absenteeism. * An item that uses a reverse scoring system.

**Table 5:** Model fit indices of the M-SVEST-R and the original SVEST-R.

Testing Paremeters	Model 1	Model 2	Model 3	Model 4	Original SVEST-R
Chi-square	1642.3	1340	1062.1	797	1555.6
df	524	498	428	418	524
*p*-value	<0.0001	<0.0001	<0.0001	<0.0001	<0.0001
RMSEA (95% CI)	0.078 (0.074, 0.082)	0.069 (0.65, 0.074)	0.065 (0.061, 0.07)	0.05 (0.044, 0.055)	0.079
CFI	0.838	0.888	0.907	0.946	0.821
TLI	0.816	0.866	0.892	0.935	-
SRMR	0.131	0.128	0.058	0.055	0.091
AIC	31,829	31,525	28,348	28,120	
BIC	31,901	31,606	28,506	28,200	

DOF: degree of freedom; RMSEA: root mean square error of approximation; CFI: comparative fit index; TLI: Tucker–Lewis index; SRMR: standardized root mean squared residual; CI: confidence interval; AIC: Akaike information criterion; BIC: Bayesian information criterion. For model 4, the distress factor combined psychological and physical distress, and the negative outcomes factor combined turnover intention and absenteeism.

**Table 6:** Raykov’s rho of the M-SVEST-R and the original SVEST-R.

	Raykov’s Rho				
Domains	Model 1	Model 2	Model 3	Model 4	Original SVEST-R
Psychological distress	0.88	0.85	0.88	0.93	0.74
Physical distress	0.91	0.90	0.91		0.86
Colleague support	0.71	0.71	0.83	0.83	0.66
Supervisor support	0.70	0.66	0.80	0.77	0.8
Institutional support	0.43	0.43	0.67	0.68	0.71
Professional self-efficacy	0.85	0.85	0.85	0.87	0.8
Turnover intentions	0.89	0.80	0.89	0.90	0.84
Absenteeism	0.82	0.74	0.82		0.79
Resilience	0.87	0.81	0.82	0.81	0.72
Total scale	0.78	0.75	0.83	0.83	0.77

For model 4, the distress factor combined psychological and physical distress, and the negative outcomes factor combined turnover intention and absenteeism.

## Data Availability

The data are not publicly available due to privacy and confidentiality. However, restrictions apply to the availability of hospital data and are available from the authors with the permission of the organization.
